# Surface scan cold plasma technology for effective inhibition of *Staphylococcus epidermidis* and *Escherichia coli*

**DOI:** 10.1038/s41598-025-32902-1

**Published:** 2026-01-19

**Authors:** Veronika Loupová, Barbora Chobotská, Přemysl Menčík, Zdenka Kozáková, Jan Hrudka, František Krčma, Kristína Trebulová

**Affiliations:** 1 Faculty of Chemistry, Brno University of Technology, Purkyňova 118, 612 00 Brno, Czech Republic; 2 University of Chemistry and Technology Prague, Technická 5, Dejvice, 166 28 Praha 6, Czech Republic

**Keywords:** Cold atmospheric-pressure plasma, Plasma medicine, Unipolar microwave discharge, Bacteria inhibition, Diseases, Microbiology

## Abstract

This study examines the decontamination efficacy of cold atmospheric plasma (CAP) against *Staphylococcus epidermidis* and *Escherichia coli*, with emphasis on surface scanning parameters and resistance potential. Bacterial cultures on solid nutrient media were treated using a non-thermal microwave plasma torch operated with high-purity argon (4.6) at 5 Slm and 12–13 W. Treatment was systematically varied in scanning direction, speed, and duration. Results indicate that exposure time was the main factor influencing bacterial inhibition, whereas direction and speed exerted only minor effects. *E. coli* exhibited greater susceptibility compared to *S. epidermidis*. Repeated exposure experiments revealed no evidence of resistance development.

## Introduction

Antibiotic resistance is a growing global issue, making it an imperative to explore alternative antimicrobial methods^1^. Cold atmospheric plasma (CAP) has emerged as a promising solution, offering effective decontamination without microbial resistance^1,2^. Thanks to its potent antimicrobial effects, cost-effectiveness, and low environmental footprint, CAP has become a promising solution for a wide range of applications^3,4^. Operating at temperatures below 40 °C, CAP is suitable for biomedical applications, including the direct treatment of living tissue and the sterilization of heat-sensitive instruments^5^.

The process of microbial cell inactivation, running under specific conditions, includes various phenomena. CAP is an ionized gas consisting of electrons, ions, excited atoms and molecules, and reactive species^6^. In argon-based microwave discharges, energetic electrons collide with argon atoms, producing excited and metastable argon states (Ar*) that transfer their energy to ambient air molecules at the plasma‑gas interface^7,8^. These secondary reactions lead to the formation of reactive oxygen and nitrogen species (RONS), including O, OH, NO, and O_2_^−^ radicals, as well as longer-lived species such as O_3_ and H_2_O_2_^9^.

Reactive oxygen and nitrogen species generated by CAP can be divided into short and long‑lived forms, each contributing differently to microbial inactivation^10^. Among the reactive oxygen species, hydroxyl radicals (·OH) exhibit the highest oxidative potential, rapidly damaging cell membranes, lipids, and DNA, while atomic oxygen (O) displays similar but slightly less aggressive reactivity^11,12^. Singlet oxygen (^1^O_2_), although less reactive, can interact with unsaturated lipids and proteins and acts as a signalling molecule within biological systems^11,13^. Hydrogen peroxide (H_2_O_2_), a long-lived and electrically neutral molecule, can easily penetrate cell membranes via aquaporins, sustaining oxidative stress even after plasma exposure^11,13^. The superoxide anion (O_2_^−^) has a relatively long lifetime and limited membrane permeability, but it plays a crucial role as a precursor of other reactive species. Together with nitric oxide (NO), it forms peroxynitrite (ONOO^−^), a highly reactive oxidizing and nitrating agent capable of inducing lipid peroxidation, protein denaturation, and DNA strand breaks^11,13,14^. Other nitrogen oxides (NO_x_) further contribute to oxidative stress and the disruption of cellular components^4^. The synergistic action of these short- and long-lived species creates complex oxidative and nitrosative conditions that ultimately result in irreversible cell inactivation.

The concentrations of short and long-lived RONS generated by CAP typically depend on the applied exposure time of the plasma jet. Longer treatment durations allow for more RONS to react with the treated surface over time and enhance the gradual accumulation of long‑lived species such as H_2_O_2_ or NO_2_^−^, whereas faster or slower scanning can influence the local flux of short-lived species to the surface^10^.

CAP also generates a localized electric field that affects cell permeability. Unlike direct electric field treatments, which rupture membranes at the poles, CAP distributes membrane damage evenly, preventing localized ruptures^15,16^. Charged particles further contribute to microbial inactivation by increasing electrostatic pressure on cell surfaces, leading to ruptures^17^.

While CAP slightly raises temperature of the treated surface, it does not increase it above 40 °C, thereby minimizing its impact on microorganisms. Instead, its antimicrobial efficacy relies on reactive particle generation. CAP also emits ultraviolet (UV) and vacuum ultraviolet (VUV) radiation, both of which can alter DNA and generate hydroxyl radicals. However, much of this radiation is absorbed within the plasma, making it a secondary factor in the microbial inactivation^15,17^. The combined effects of these mechanisms result in efficient decontamination^18,19^.

A number of studies highlight the potential of CAP in wound healing, particularly in treating chronic wounds like diabetic foot ulcers^2,20,21^. CAP applications are also being explored in dermatology, skincare^22,23^, and oncology^24,25^.

To assess the antimicrobial effects of CAP, two representative bacterial species were selected: *Escherichia coli* (*E. coli*) and *Staphylococcus epidermidis* (*S. epidermidis*). These microorganisms were chosen based on their clinical relevance, prevalence, and differing cell wall structures, which allow for comparison of CAP efficacy against both gram-negative and gram-positive bacteria. *E. coli* represents one of the most studied gram-negative model organisms, while *S. epidermidis* is a typical representative of gram-positive skin microbiota and is also a major opportunistic pathogen in nosocomial infections^26–31^.

*E. coli* is a facultative anaerobic, non-sporulating, gram-negative bacterium of the Enterobacteriaceae family^26,27^. *E. coli* has a rod-shaped structure (1–1.3 μm × 0.4–0.7 μm). It is often motile due to peritrichous flagella. It is found commonly, in warm‑blooded animals, food, and the environment. *E. coli* is a part of the normal microbiota; nevertheless, it can also show pathogenic effects^26^. Pathogenic *E. coli* strains cause various diseases, including diarrheal infections and urinary tract infections. Some strains can also cause meningitis or sepsis^16,17^. Intestinal pathotypes include enteropathogenic (EPEC), enterohemorrhagic (EHEC), enterotoxigenic (ETEC), enteroaggregative (EAEC), enteroinvasive (EIEC), and diffusely adherent (DAEC) *E. coli*^27^. These diarrheagenic strains differ in their virulence mechanisms, host interactions, and symptoms^32,33^.

*S. epidermidis* is a gram-positive, facultatively anaerobic bacterium classified as a coagulase‑negative staphylococcus^28^, forming non-motile, clustering cocci (0.5–1.5 μm). It is commonly found on human skin and mucous membranes, particularly in the armpits, nasal cavities, and scalp^28,30^. Specific strains produce slime, thereby making colonies appear transparent and sticky^29^. Once considered harmless, *S. epidermidis* is now recognized as an opportunistic pathogen, frequently linked to nosocomial infections. It is associated with contaminated medical devices, particularly catheters, which contribute to increased patient mortality^29–31^. The bacterium is also implicated in surgical site infections, prosthetic joint infections, and eye infections caused by contaminated contact lenses^28^. Additionally, *S. epidermidis* is prevalent in the oral cavity, where it may contribute to dental conditions like periodontitis, pulpitis, and dry socket^29^. Its widespread colonization and virulence factors make it a significant source of infections^28^.

CAP can be generated by various plasma sources. This study deals with the CAP generated by the low-temperature microwave discharge with direct gas flow (argon). The design of this device is legally protected and was created by František Krčma (Czech Republic) and Todor Bogdanov (Bulgaria)^34^. The potential of microwave plasma sources, although offering promising characteristics such as stable operation and efficient generation of RONS, remain relatively less explored compared to other types of discharges. Therefore, this study aims to contribute to a deeper understanding of their potential in antimicrobial applications^35^.

Current literature lacks detailed evaluations of how scanning direction and movement speed of CAP jets influence antimicrobial outcomes. This study addresses this gap by systematically analysing these parameters using a programmable scanning system, offering valuable insights for future biomedical implementations.

Specifically, the study aims to optimize the 3D movable mechanism of the unipolar microwave torch (Surfayok) and evaluate its effect on *Escherichia coli* and *Staphylococcus epidermidis*. The implementation of this movement system enables precise control over the plasma exposure area and ensures reproducible treatment of well-defined surfaces, such as the surface of a Petri dish used in this study.

## Experimental section

### CAP source–unipolar microwave torch (Surfayok)

**Fig. 1:**
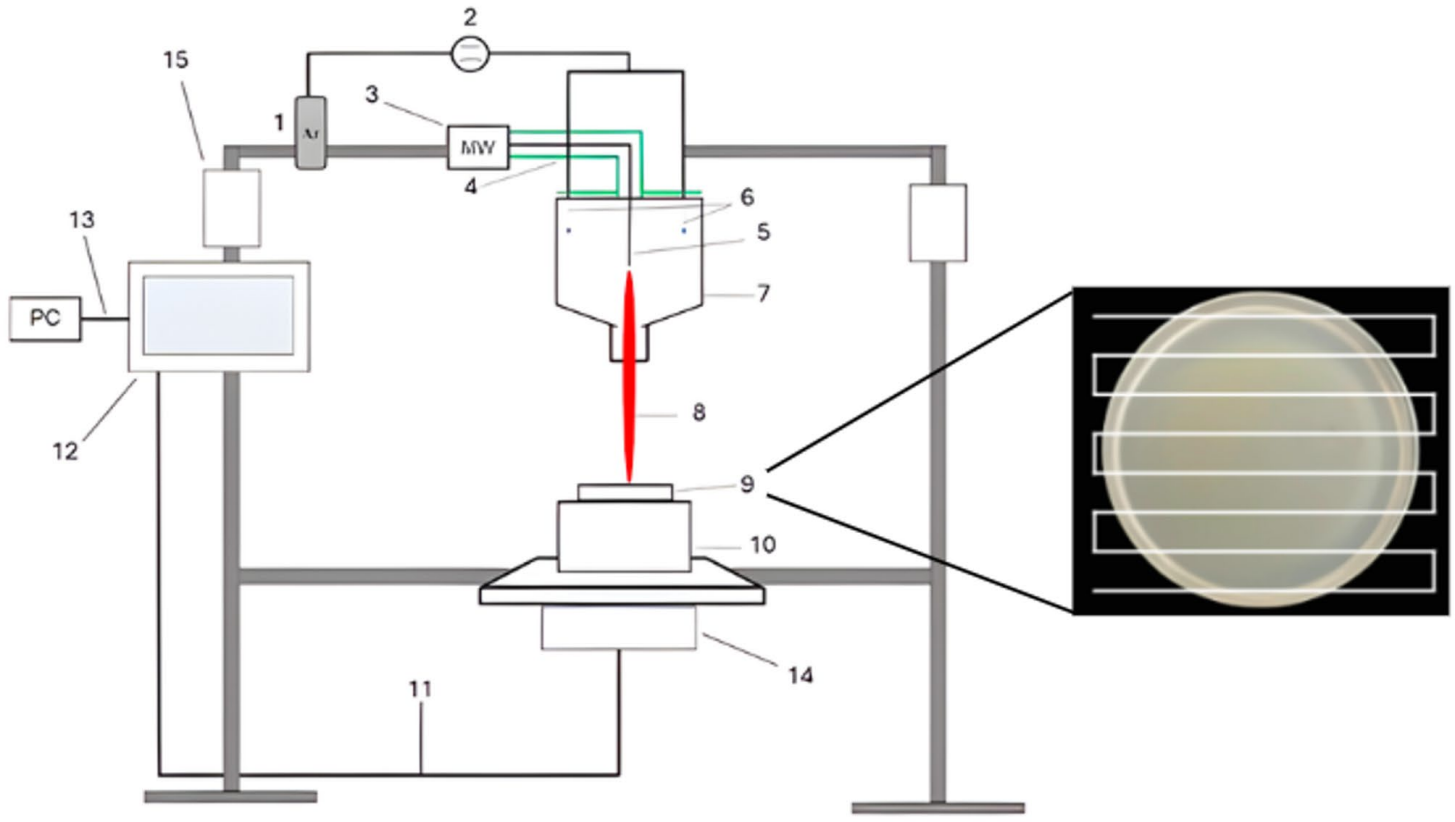
Experimental setup of the unipolar microwave (MW) torch: 1—argon source, 2—mass flow controller, 3—MW source, 4—MW coaxial cable, 5—MW antenna, 6—direct argon supply, 7—resonance cavity, 8—plasma beam, 9—Petri dish with culture, 10—sliding Petri dish holder, 11—cable, 12—display of 3D movable system, 13—USB cable, 14, 15—step engine of 3D movable system. The inset illustrates the linear scanning trajectory programmed in the 3D movable system, which enables uniform plasma exposure across the entire Petri dish surface.

Microwaves were generated using a microwave generator (Sairem, GMS 200 W) operating at a frequency of 2.45 GHz. Microwaves were launched into a resonant cavity, where they were distributed throughout the entire cavity via a microwave antenna. This setup enabled the ionization of argon atoms. Argon was supplied to the cavity in parallel with MW antenna through a mass flow controller (Bronkhorst FG201-CV), which was set to a flow rate of 5 Slm. Inside the resonant cavity, ionized gas − plasma was formed and subsequently expelled from the cavity through an outlet (8 mm in diameter). The plasma was manifested visible in the form of filaments, which were in direct contact with the treated Petri dish (see Fig. 1).

A 3D movable system controlled by PC software was used for a reproducible scanning treatment of microbial cultures on the Petri dish. The distance between two parallel scanning lines was 3 mm, while the area covered by the plasma plume was approximately 5‒6 mm^2^. During the automated scanning, the plasma torch was programmed to move slightly beyond the Petri dish edges in each pass, ensuring complete surface exposure (Fig. 1). As a result, the effective surface coverage was 100%, independent of the scan speed. Therefore, the scan speed influenced only the local exposure time per unit area, while the total treated surface area remained constant for all tested conditions.

### Preparation of agar-based biopolymers for colorimetric detection

Agar-based biopolymers were prepared by dissolving 2.5 g of agar in 100 mL of distilled water. The mixture was heated to 85 °C under continuous stirring until fully homogenised, then allowed to cool to 60 °C. At this temperature, the selected colorimetric reagents were incorporated to enable qualitative detection of reactive species generated during CAP treatment. The following compounds were added per 100 mL of agar solution: indigo dye (0.001 g) for ozone detection, potassium iodide (0.5 g) with starch (0.4 g) for non-specific ROS detection, and Griess reagent powder kits (Merck; added according to the manufacturer’s instructions) for nitrite detection. After thorough mixing, the agar solutions were poured into Petri dishes and allowed to solidify. The prepared agar-based biopolymers were subsequently used for CAP exposure experiments.

### Optical emission spectrometry

**Fig. 2:**
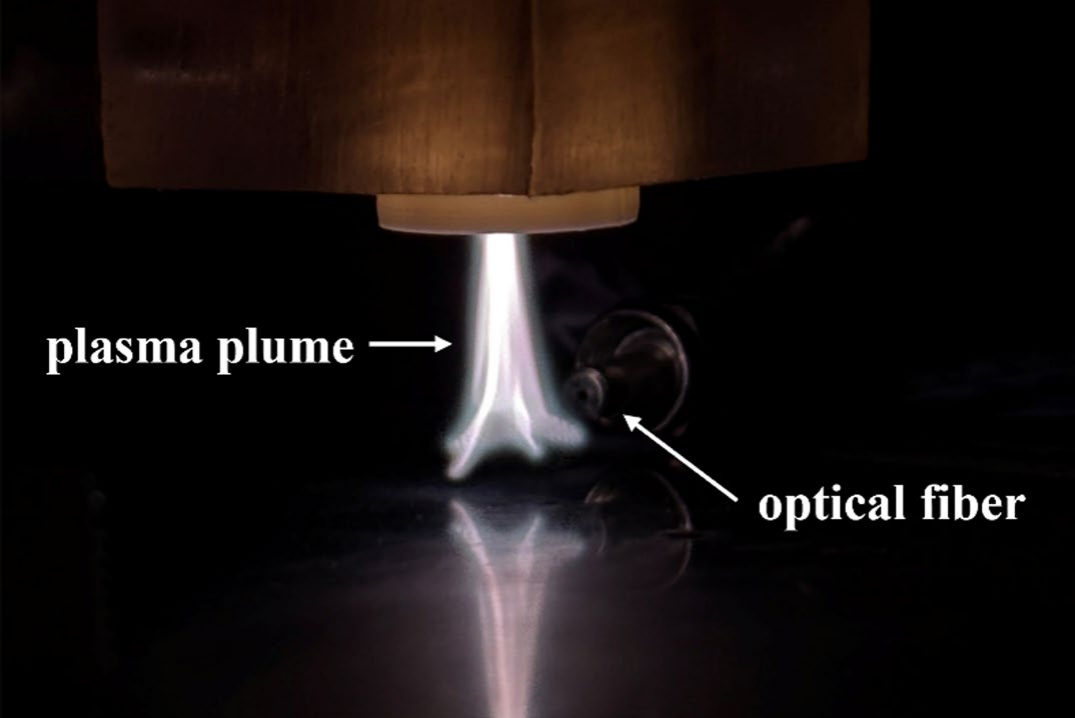
OES set-up for the recording of emission spectra from the plasma plume.

The light emitted by the plasma plume was collected through a quartz optical fibre (Fig. 2) and analysed by means of a spectrometer (Oceanoptics HR4000). The spectra were averaged over 10 acquisitions of 30 ms of exposure to analyse the whole spectrum (330–790 nm) and for 2000 ms to zoom on the UV region (330–450 nm) and to register emission of less abundant excited species.

### Preparation of microorganisms

To test the antimicrobial activity of the unipolar microwave torch (Surfayok), bacterial strains of *Escherichia coli CCM 3954* and *Staphylococcus epidermidis CCM 4418* were used; both were obtained from the Czech Collection of Microorganisms (CCM), Masaryk University, Brno, Czech Republic. *Escherichia coli* was cultured in LB (Lennox Broth) and *Staphylococcus epidermidis* in BHI (Brain Heart Infusion) liquid medium, both at 37 °C for 20 h under aerobic conditions. From the resulting 20-h cultures, bacterial suspensions with a final concentration of 10^6^ CFU/mL were prepared by appropriate dilution in sterile LB medium for *E. coli* and in BHI medium for *S. epidermidis*.

The optical density of the diluted cultures was measured at a wavelength of 630 nm using a UV–VIS spectrophotometer VWR UV-1600PC. The corresponding cell concentrations were calculated based on the measured absorbance values using organism-specific calibration equations (*y* = 2.186·*x* for *S. epidermidis* and *y* = 2.853·*x* for *E. coli*, where *x* is expressed in 10⁹ CFU/mL). Subsequently, a series of tenfold dilutions was prepared to obtain cultures with final concentrations down to 102 CFU/mL.

The sterile nutrient agar (LB or BHI) was evenly poured into sterile polystyrene Petri dishes with an inner diameter of 55 mm and allowed to solidify under sterile conditions. Then, aliquots of 50  µL of the appropriately diluted suspension was inoculated onto the surface of the solidified agar plates. Immediately after inoculation, the plates were exposed to the plasma under a continuous gas flow of 5 standard litres per minute (5 Slm).

### Treatment of microorganisms

#### E. coli

It is important to note that the initial optimization of the setup was performed using *E. coli*. This included adjusting the movement of the plasma torch and determining the optimal treatment durations and speeds to achieve maximum surface decontamination. Consequently, fewer measurements were performed for *S. epidermidis*.

The Petri dishes inoculated with *E. coli* were treated with plasma immediately after inoculation. The distance from the treated agar surface was set so that the plasma tip was just in contact with the surface. Three main treatment parameters were investigated: treatment time (1 min, 2 min, 5 min, 8 min), treatment orientation (parallel, perpendicular), and treatment speed (8.3–9.4 mm/s). As mentioned above, the microwave power was set to 12 W, and the argon gas flow rate was maintained at 5 Slm. All measurements were performed in triplicates for each concentration (10^6^, 104 CFU/ml) and each treatment condition (direction, duration, and speed). For each measurement series, untreated controls were included, and an additional argon-only control was performed for the 8 min exposure.

After each treatment, all Petri dishes were incubated in a thermostat at 37 °C for 24 h.

In addition, repeated treatment experiments were performed, in which an inoculum was prepared from a previously treated culture and incubated at 37 °C with shaking for 24 h. The resulting culture was subsequently diluted and subjected to the same treatment procedure as described above.

#### S. epidermidis

The Petri dishes with *S. epidermidis* culture were also treated with CAP immediately after inoculation. In this case, only two treatment aspects were studied: treatment duration (2 min, 5 min, 8 min) and treatment speed, again assessed for the 2 min treatment, only. The countable controls at concentrations of 102 and 103 CFU/ml were included. Argon-treated controls were performed only for the longest exposure time (8 min). The power and gas flow rate settings were identical to those used for *E. coli*.

Repeated treatment experiments were also conducted with *S. epidermidis*. The inoculum was prepared from previously treated cultures and incubated for four hours in a shaking thermostat, since the exponential growth phase for this strain begins after four hours. Then, the inoculum was diluted to the target concentrations (10^6^, 103, and 102 CFU/ml), and the Petri dishes were treated as described previously. Repeated treatments were performed over four consecutive days, where inoculum was prepared each day from the culture treated the previous day.

### pH measurement of agar surfaces

The pH of the agar surface was measured for both LB and BHI agar plates prepared as described above. For each medium, three conditions were evaluated: untreated control, plasma-treated for 5 min, and plasma-treated for 8 min. Surface pH measurements were performed immediately after treatment using a contact pH electrode (Mettler Toledo SevenEasy), which allows direct measurement on semi-solid surfaces.

### Data processing and evaluation

After the incubation period, all the Petri dishes were photographed. Colony counts were evaluated in collaboration with HexTech Research s.r.o. using the Aurora software, developed by Ing. Jan Hrudka^36^. This program uses machine learning and artificial intelligence to determine colony counts. Based on the collected data, graphs were generated to visualize the reduction in colony counts compared to those in untreated controls for each treatment condition. For clarity, all concentrations were recalculated and normalized to a reference concentration of 10⁶ CFU/ml for consistent comparison across the samples. Results were also statistically evaluated using the software Statistica. Standard statistical tools were used to evaluate the significance of achieved results. According to the data characteristics, statistical analysis was performed using t-tests and one-way ANOVA, with a significance level of α = 0.05.

## Results and discussion

### Optimization of the treatment conditions

The applied power of the microwave plasma source (Surfayok) was set to 12 W in continuous mode. This value was selected based on previous results reported by Trebulová et al.^35^, which demonstrated a clear improvement in antimicrobial efficacy compared to operation at 9 W. Specifically, the inhibition zone area increased from approximately 4.5–5 cm^2^ at 9 W to 6–7 cm^2^ at 12 W across various exposure regimes (30 s, 3 × 10 s, 60 s, 2 × 30 s), corresponding to an average enhancement of about 25%. The higher power thus enabled more effective and uniform bacterial inactivation within shorter exposure times.

Our preliminary tests further confirmed that 12 W ensured a stable plasma discharge without visible thermal damage to the agar surface, representing a suitable compromise between antimicrobial efficiency, plasma stability, and sample safety.

Argon was selected as the working gas due to its ability to sustain a stable, low-temperature microwave discharge. As an inert monoatomic gas with a moderate ionization energy (15.76 eV), argon facilitates efficient plasma generation at relatively low power while maintaining a non-thermal regime. Although helium is also frequently used in cold plasma systems due to its low gas temperature and high diffusion coefficient, its high ionization energy (24.6 eV) requires increased power input for ignition, resulting in reduced plasma density and higher operational costs. Conversely, molecular gases such as oxygen or nitrogen, despite their potential to enhance RONS formation, tend to increase gas temperature due to energy consumption for rotational and vibrational energy transitions. Therefore, argon represents an optimal compromise between plasma stability, antimicrobial efficacy, and thermal safety, making it particularly suitable for biomedical-oriented applications.

To determine an appropriate treatment distance, agar-based biopolymers enriched with indigo blue dye were employed as surface indicators of reactive oxygen and nitrogen species, predominantly ozone. Indigo contains a carbon–carbon double bond that undergoes stoichiometric oxidation by ozone, resulting in dye decolourisation^37,38^. Although other plasma‑generated ROS may contribute to oxidation, the reaction remains a reliable qualitative marker of ROS–surface interactions.

Three distances within and above the plasma discharge were examined: (i) the central region of the discharge in direct contact with the surface, (ii) the tip of the discharge—later used for all antimicrobial assays, and (iii) the central portion of the plasma plume positioned 10 mm above the surface. The indigo-based visualisation revealed that the most pronounced and uniform decolourisation occurred when only the plume tips interacted with the surface, indicating the highest concentration of RONS at this position. This observation is consistent with previously reported distance-dependent plasma effects^39^ and correlated with the highest antimicrobial efficacy. Therefore, this distance was selected for subsequent decontamination experiments.

### Colorimetric detection of RONS in plasma-treated surfaces

Agar-based biopolymer indicators were also used to assess the types of reactive species generated during plasma scanning over the Petri dish surface. Because optical emission spectroscopy alone does not capture RONS behaviour at the plasma‑surface interface^40^, this colorimetric approach was used to visualise the spatial distribution of reactive species on treated agar surfaces.

**Fig. 3:**
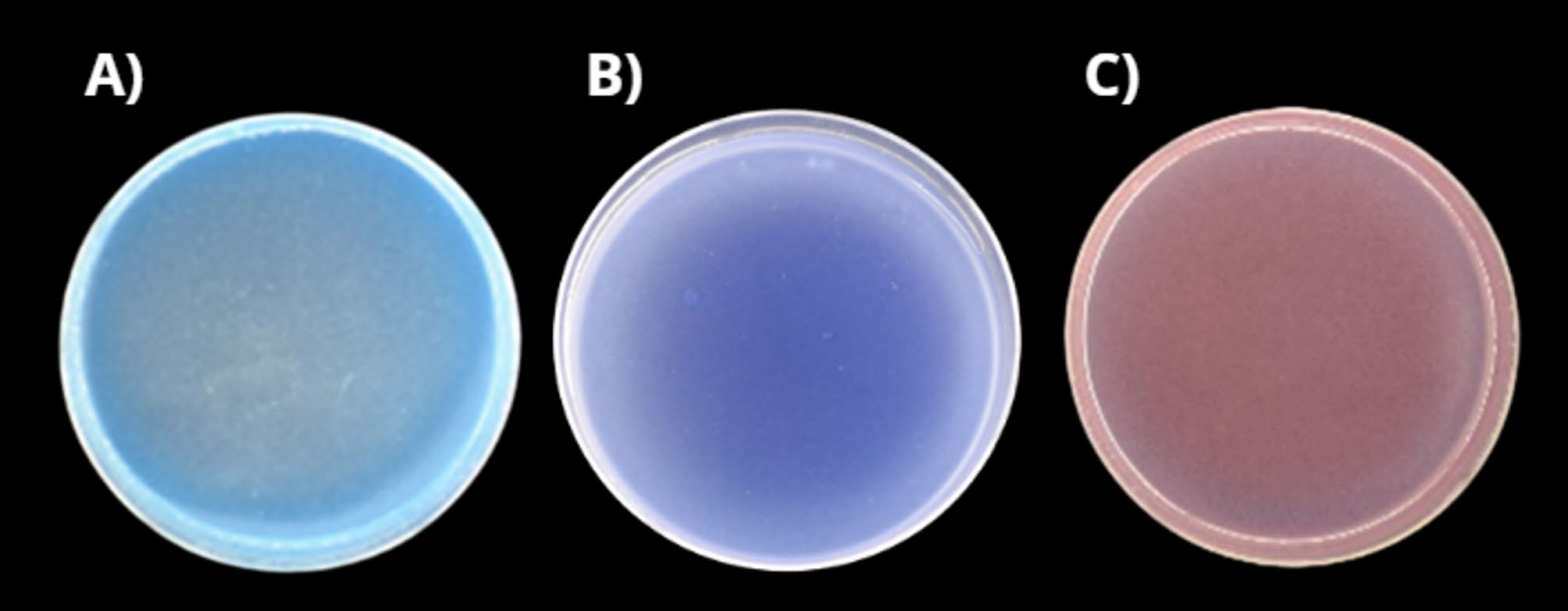
Detection of RONS by agar-based biopolymers **A** detection of ozone by indigo, **B** detection of ROS by KI-starch admixture, **C** detection of nitrites by Greiss reagent kit.

Indigo, as previously mentioned, was employed primarily for the detection of ozone. As can be seen in the Fig. 3a, almost the whole area of the indigo‑enriched biopolymer was completely discoloured after 500 s of scanning, with only the blue edges remaining.

The KI‑starch system was used to detect ROS. In this case, non-specific oxidation of iodide to iodine occurs in the presence of ROS, and the formed iodine subsequently interacts with starch to produce a dark blue complex, as shown in Fig. 3b. In the absence of reactive species, this agar-based biopolymer remains colourless. As shown in Fig. 3b almost a whole surface of the biopolymer was homogeneously coloured after 300 s of the CAP treatment, but the edges stayed transparent.

Furthermore, the presence of RNS was evaluated using the Griess reagent kit. While nitrates were not detected, nitrites were confirmed by a colour change from colourless to pink, homogeneously distributed over the whole agar plate, including the edges (Fig. 3c). The plasma treatment duration for RNS indicator assays was 300 s.

### Optical emission spectrometry

**Fig. 4:**
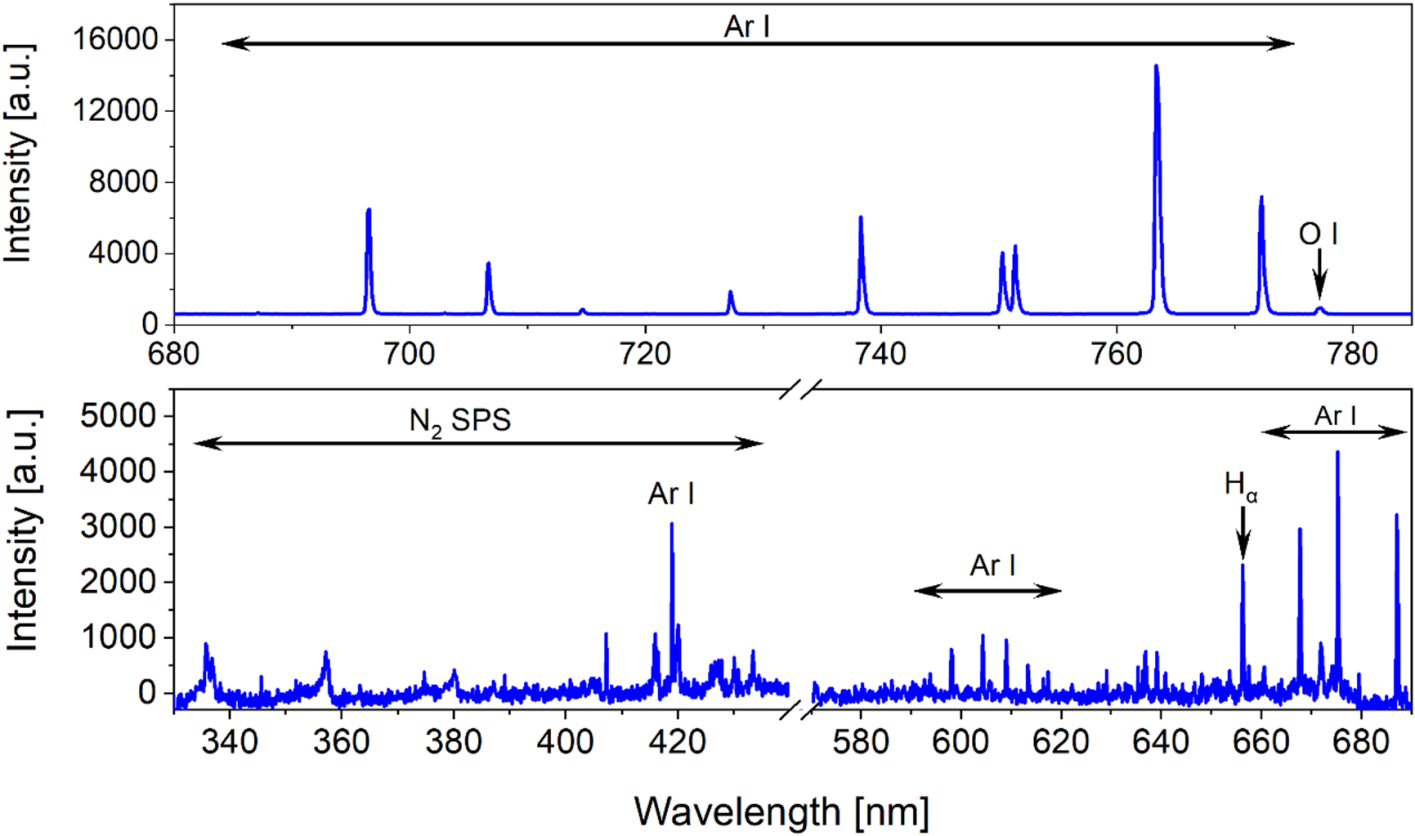
Optical emission spectrometry data for Surfayok 12 W.

In the recorded emission spectra (Fig. 4), the most prominent features correspond to argon atomic lines, which represent the dominant emission of the discharge. The first spectrum, covering the 680–790 nm region, was acquired using a short acquisition time of 30 ms because the high intensity of argon emission led to detector saturation during longer acquisition. In addition, the second spectrum (330–690 nm) was measured with a longer exposure time (2000 ms), which was necessary to capture the comparatively weak emission from nitrogen and hydrogen. The detection of these excited species provides indirect evidence for the generation of RONS, as the excited molecules can undergo further ionization or dissociation processes. This is supported by the observed second positive system of nitrogen (N_2_ SPS) in Fig. 4, as well as the presence of atomic hydrogen (H_α_) at 656 nm or triplet oxygen (O) emission at 777 nm, both of which indicate interactions between the plasma and ambient air^41,42^.

### Effect of CAP treatment on agar pH

The surface pH of the agar plates was assessed using a contact electrode (Mettler Toledo SevenEasy) to determine whether CAP treatment induced measurable acidification of the medium. Only minimal pH changes were observed for both LB and BHI agar, with values remaining within the typical variability of untreated plates. These findings indicate that the antimicrobial effects observed in this study cannot be attributed to pH alterations of the agar surface.

### Influence of treatment direction

**Fig. 5:**
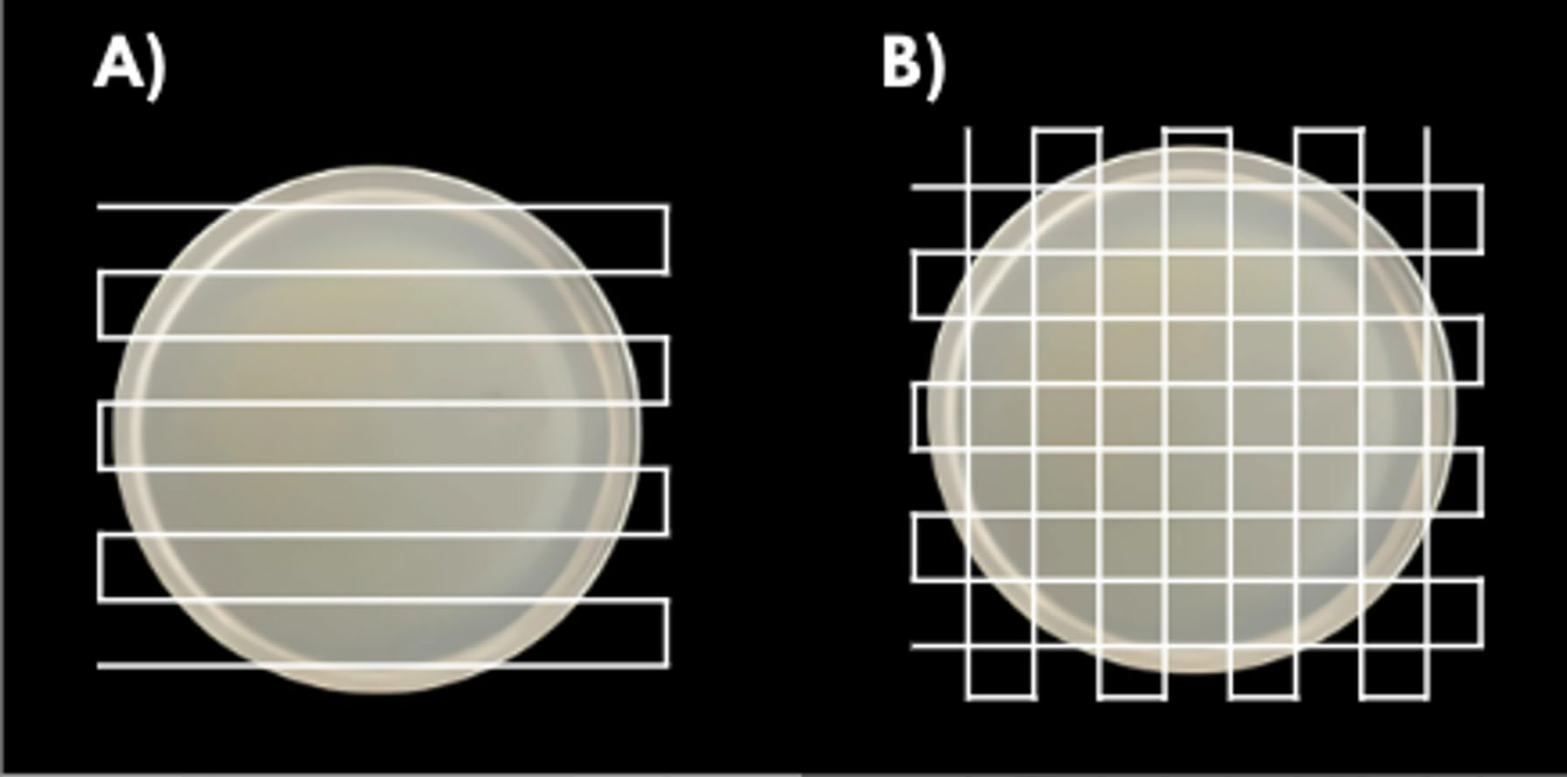
Treatment type: **A** parallel, **B** perpendicular.

The effect of different displacement patterns of the plasma torch on the decontamination of the treated surface was investigated. The treatment was performed exclusively on bacterial cultures of *Escherichia coli*. Two types of treatment protocols were employed. In the first one, the plasma jet moved along the same linear path over the inoculated agar plate for two minutes (treatment type: parallel). Using the second approach, the plasma jet was applied for one minute in one direction, followed by a 90° rotation of the Petri dish and a subsequent one-minute treatment in a direction perpendicular to the initial path (treatment type: perpendicular) (see Fig. 5).

**Fig. 6:**
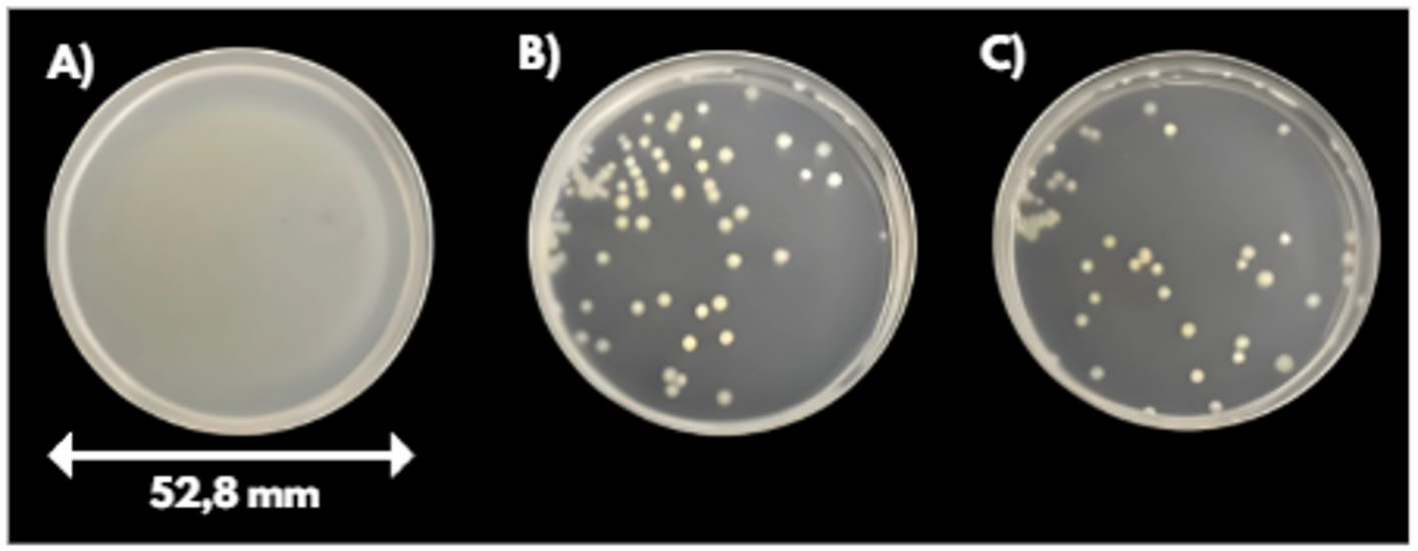
Visualization of E. coli bacterial cultures with an initial concentration of 10^6^ CFU/ml after 24 h incubation: **A** Control, **B** Perpendicular treatment, **C** Parallel treatment.

**Fig. 7:**
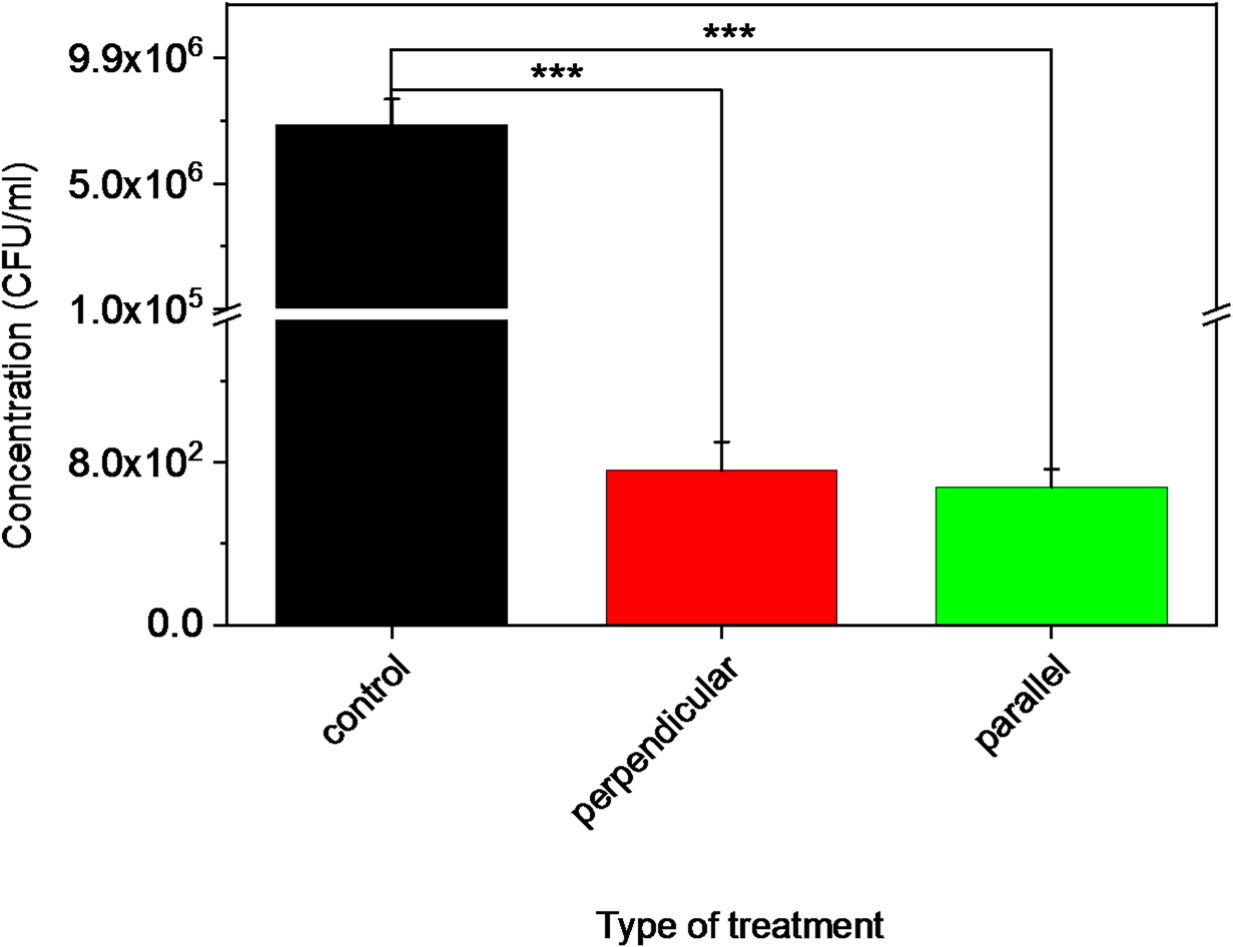
Bacterial reduction (CFU/mL) after plasma treatment using parallel and perpendicular scanning directions.

Compared to the untreated control, both treatment protocols resulted in a highly significant reduction in viable cell counts (t-test, t (5) = 14.30, *p* = 0.00003, ***), corresponding to a ~ 4 log10 reduction (Figs. 6 and 7). Corresponding numerical data are shown in Table 1 in the Appendix. Statistical analysis further revealed no significant difference between the two treatment types (t-test, t (13) = − 0,85, *p* = 0.41), indicating that both approaches can be considered equally effective under the tested conditions.

To the best of our knowledge, the impact of scanning direction in CAP-based antibacterial treatments has not been systematically investigated in the existing literature. Our findings suggest that within the tested configurations, scanning direction had no significant effect on bacterial reduction, which may simplify future applications requiring automated plasma movement. Moreover, the lack of directional dependence indicates that the treatment could also be effectively applied manually, for example by a clinician, without compromising efficacy.

In our setup, the entire surface of the Petri dish was covered by the automated scanning trajectory, ensuring uniform direct exposure to the plasma plume. Under these conditions, direct plasma‑cell interactions are expected to dominate the inactivation process, as the short-lived RONS generated in the gas phase (e.g. ·OH, O, ^1^O_2_) have very limited diffusion ranges in agar. Although long-lived species may contribute to additional post-treatment effects, the absence of directional differences supports the interpretation that direct exposure plays the primary role under the tested conditions.

### Influence of the treatment time

**Fig. 8:**
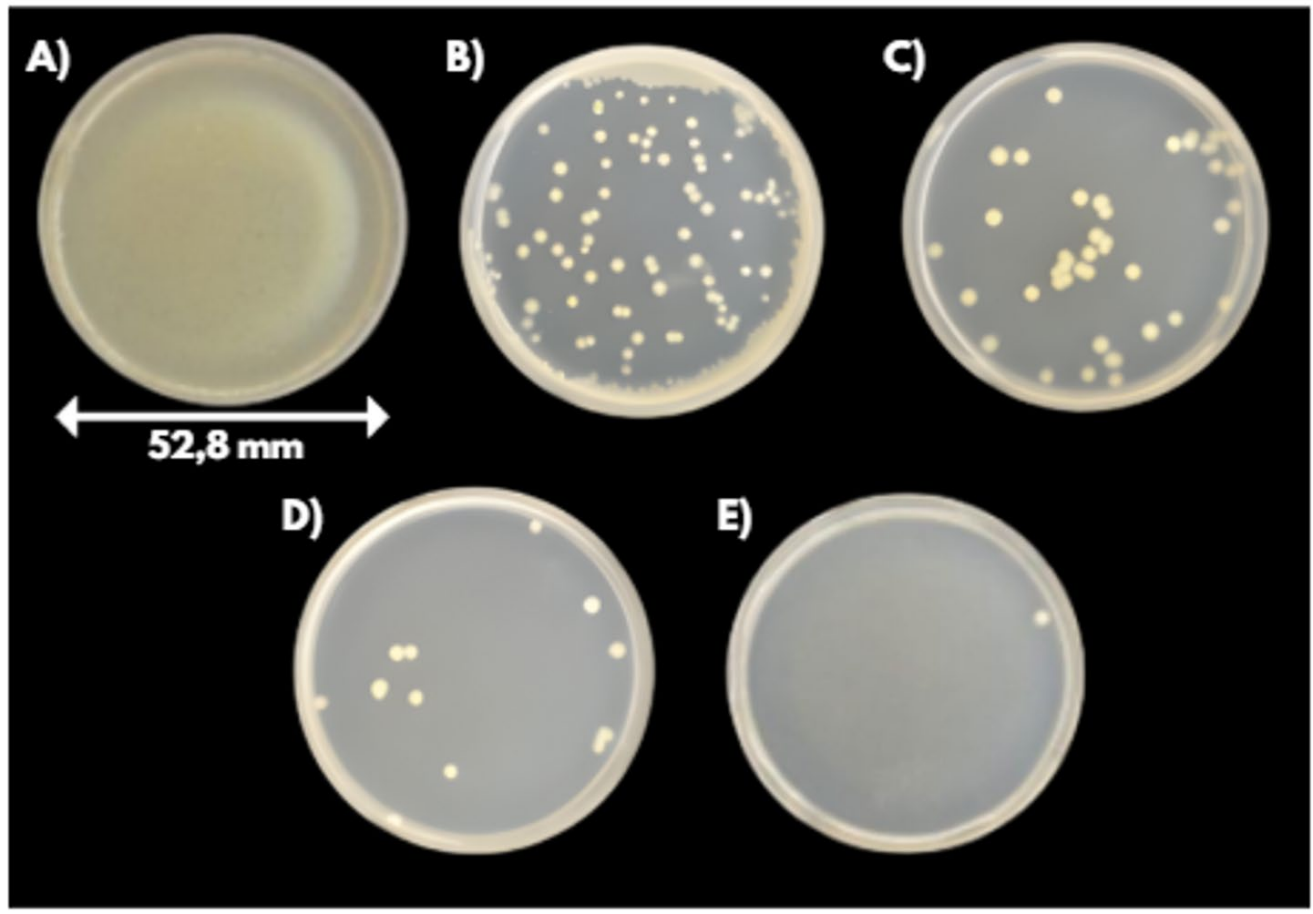
Visualization of the inhibitory effects at different treatment times for E. coli with an initial concentration of 10^6^ CFU/ml: **A** Control (10^6^ CFU/ml), **B** 1 min treatment, **C** 2 min treatment, **D** 5 min treatment, **E** 8 min treatment.

**Fig. 9:**
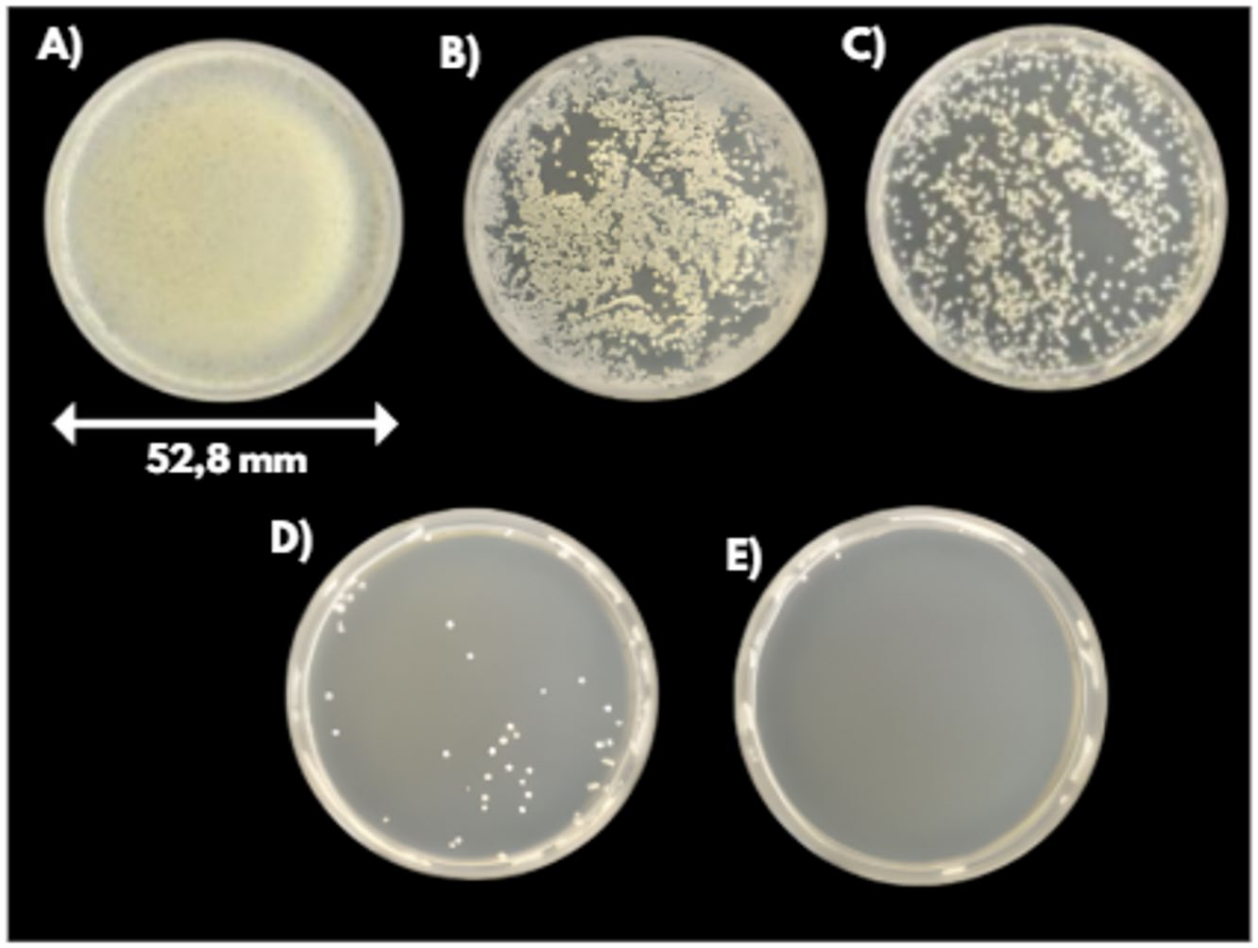
Visualization of the inhibitory effects at different treatment times for S. epidermidis with an initial concentration of 10^6^ CFU/ml: **A** Control (10^6^ CFU/ml), **B** 1 min treatment, **C** 2 min treatment, **D** 5 min treatment, **E** 8 min treatment.

The effect of treatment time was also investigated using four different treatment times: 1, 2, 5, and 8 min. Since no significant differences were observed, parallel scanning was applied in this case and continued to be used thereafter, as it provided a simpler and more convenient procedure. All results are expressed relative to the untreated controls, as no significant difference was observed between the untreated plates and those exposed to argon gas alone for 8 min. This confirms that the gas flow itself did not exhibit any inhibitory effect and therefore cannot be considered a contributing factor to the observed bacterial reduction. Figures 8 and 9 showing the agar plates after 24 h of incubation indicate that longer treatment times resulted in increased decontamination in both bacterial species tested.

**Fig. 10:**
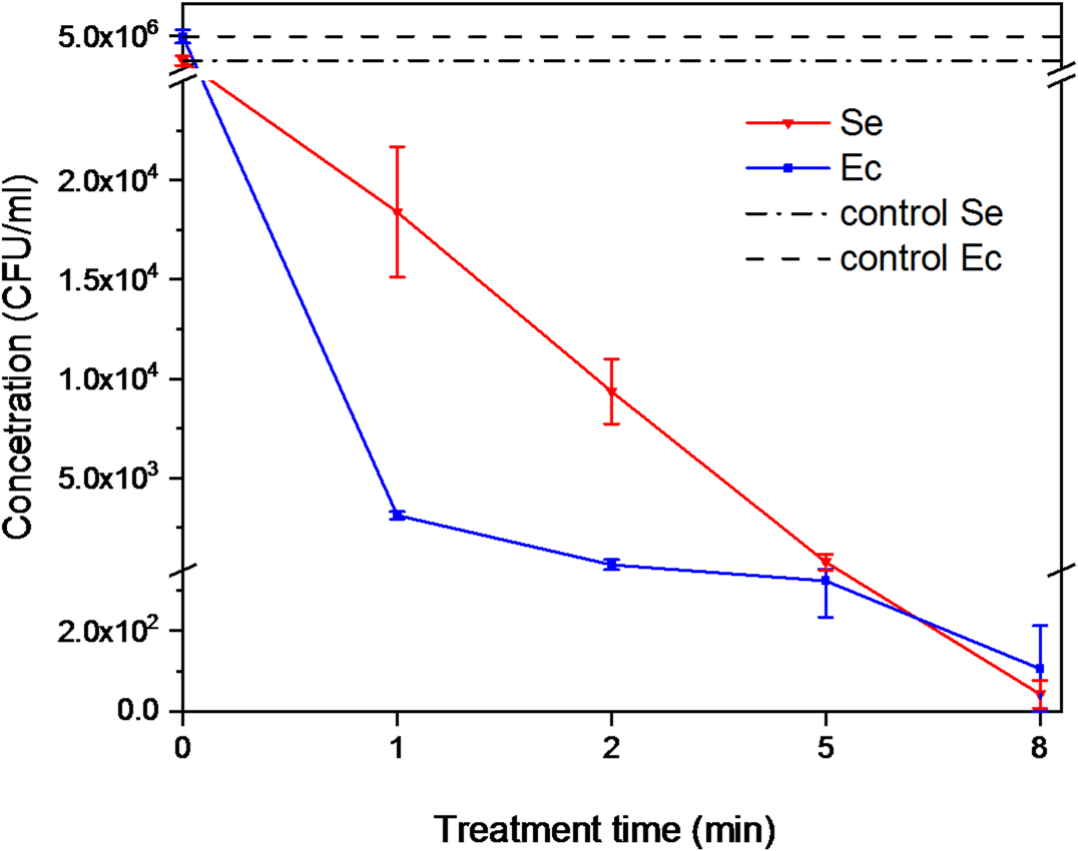
Bacterial reduction dependence on the treatment time; the red curve represents S. epidermidis, the blue curve represents E. coli, and the black curves indicate control values for both bacterial strains.

At shorter treatment times (one and two minutes), a greater reduction in colony counts was observed for the Gram-negative bacterium *Escherichia coli* (Ec) compared to the Gram-positive *Staphylococcus epidermidis* (Se) (see Fig. 10). This finding is consistent with the known structural differences between these groups, as Gram-negative bacteria possess a thinner peptidoglycan layer in their cell walls, making them more susceptible to plasma-induced damage^43^.

Notably, treatments lasting five and eight minutes resulted in comparable levels of bacterial decontamination for both species (see Fig. 10). At these exposure times, the plasma-generated RONS are present in high concentration and for a long time sufficient to ensure effective decontamination of the targeted area.

These results also reflect how different types of reactive species participate in the inactivation process. At short exposure times, highly reactive and short-lived species (such as ·OH or O) act almost immediately and can more easily disrupt the thinner outer structures of *E. coli*. In contrast, the more robust peptidoglycan layer of *S. epidermidis* requires longer exposure for sufficient levels of both short- and long-lived species to accumulate. With increasing treatment duration, the continued generation of reactive species reduces this initial difference, which explains why both microorganisms reach comparable levels of reduction at five- and eight‑minute treatments.

The corresponding log reduction values (CFU/ml) for different treatment durations are detailed in Table 2 in the Appendix, showing a logarithmic decrease ranging from 3 to 5. The highest reduction was achieved for *S. epidermidis* after the longest (8 min) treatment; however, when taking the standard deviation into account, the values can be regarded as comparable for both microorganisms (t-test, t (9) = 0,58, *p* = 0.58).

The findings of this study are in line with previous research on plasma-based bacterial inactivation. For instance, Prochnow et al.^44^ and Huang et al.^45^ reported similar trends in the differential sensitivity of Gram-positive and Gram-negative bacteria to non-thermal plasma treatment.

### Influence of different speed of the treatment

The effect of treatment speed was investigated while keeping the overall exposure time constant at two minutes. The plasma torch scanned across the surface at four different speeds, corresponding to one, two, five, or eight passes within the fixed time. These regimes covered a range from 8.3 mm/s (the slowest) to 69.4 mm/s (the fastest), with intermediate conditions at 16.5 and 43 mm/s. Thus, higher numbers of passes simply reflected proportionally faster scanning of the surface, without altering the total treatment duration.

**Fig. 11:**
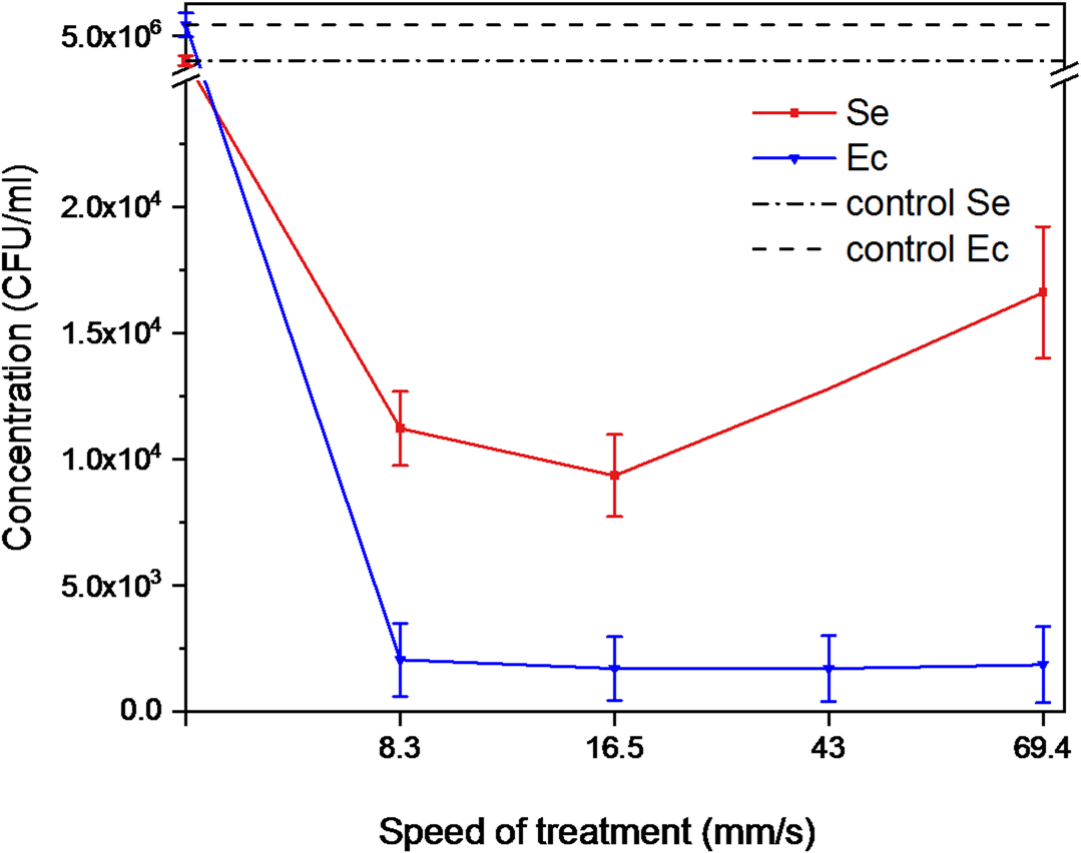
Impact of treatment speed on surface decontamination efficiency; the red curve represents S. epidermidis (Se), the blue curve represents E. coli (Ec), and the black curves indicate control values for both bacterial strains.

A graph illustrating the relationship between treatment speed and bacterial response was constructed for both strains under the study (see Fig. 11).

For the Gram-negative bacteria *E. coli*, scanning speed across 8.3–69.4 mm/s had no significant impact on colony suppression (F (3, 8) = 0.03, *p* = 0,994, ANOVA). As decontamination efficacy did not differ among speeds, only three representative conditions were selected for further tests with the Gram-positive *S. epidermidis*: 8.3 mm/s (the slowest), 16.5 mm/s (intermediate, also used in earlier experiments), and 69.4 mm/s (the fastest).

For *S. epidermidis*, speed appeared to play a more noticeable role. The fastest regime (69.4 mm/s, eight passes) resulted in slightly more survived colonies after 24 h cultivation compared to slower treatments, suggesting reduced efficacy. This trend may be linked to the thicker Gram-positive cell wall, where the very rapid scanning could limit local exposure below the threshold required for effective inactivation. However, a linear regression analysis revealed no statistically significant effect of scanning speed on decontamination efficacy (F (1, 4) = 0,38, *p* = 0.569, ANOVA), indicating that the observed trend was not significant under the tested conditions.

This observation is consistent with the general behaviour of CAP, where the overall inactivation is primarily determined by the total amount of reactive species delivered to the surface. When the entire area is scanned uniformly, this integrated exposure remains essentially the same and therefore changes in scanning speed do not substantially influence the underlying physicochemical interactions at the cell‑plasma interface.

The logarithmic reduction for both microorganisms at different speed of treatment is provided in Table 3 in the Appendix.

Overall, these results suggest that rapid scanning should be avoided in practical applications, as high speeds can compromise decontamination. While the treatment duration has been studied extensively, scanning speed as an independent parameter remains underexplored. Therefore, optimizing both movement and exposure time is essential for reliable antibacterial performance, especially against more resilient microorganisms.

### Influence of repeated treatment

Pilot experiments were conducted to determine whether bacterial resistance to the plasma treatment might develop.

#### Escherichia coli

**Fig. 12:**
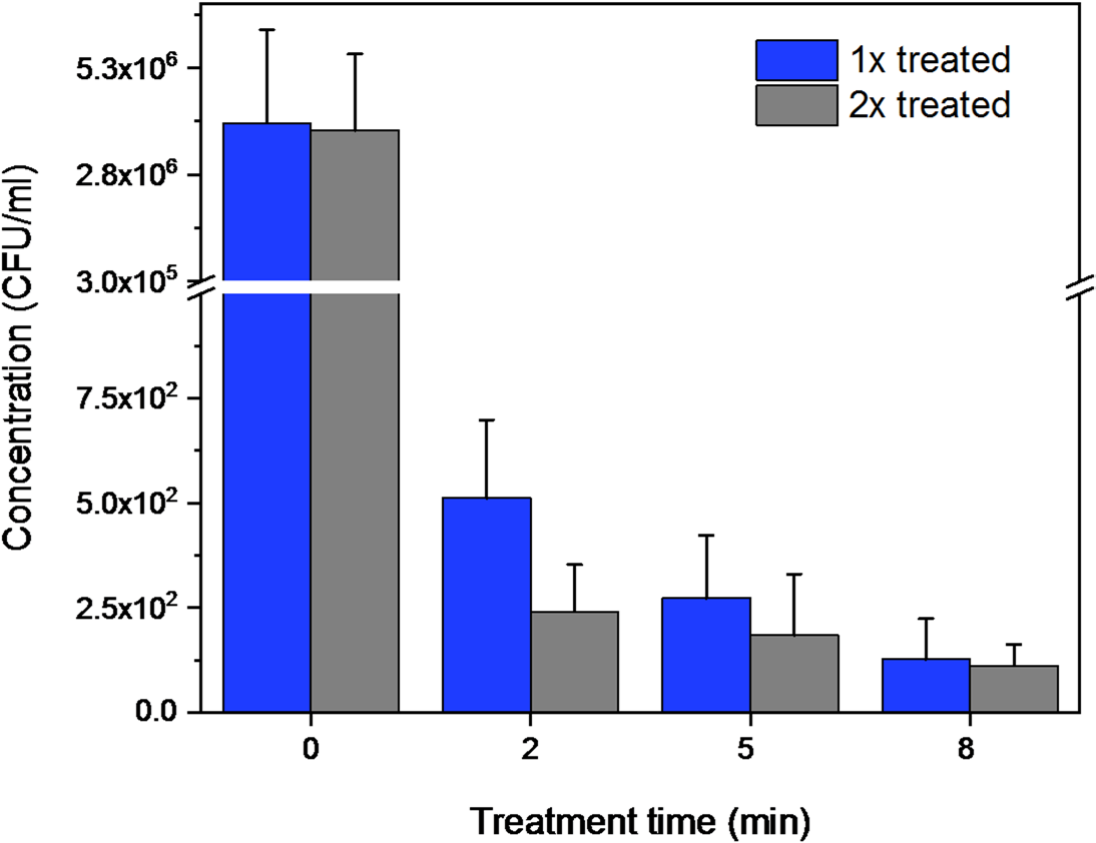
Comparison of single and repeated plasma treatment of E. coli. The blue bars represent samples treated once, while the grey bars correspond to the samples treated twice using inoculum derived from the previously treated cells.

For *E. coli*, the repeated treatment was performed only once, when a new inoculum was prepared from the previously treated cells and subsequently diluted to the required concentrations. As shown in the graph (see Fig. 12), no increase in colony growth was observed during the second treatment. On the contrary, a slight decrease in colony numbers was observed for all treatment times, indicating that no bacterial resistance to the plasma treatment was observed under the tested conditions (This is also shown in detail in Table 4, presenting the logarithmic reduction values provided in the Appendix). However, it should be noted that additional repetitions and extended exposure cycles would be necessary to draw a definitive conclusion regarding the potential development of bacterial resistance.

#### Staphylococcus epidermidis

**Fig. 13:**
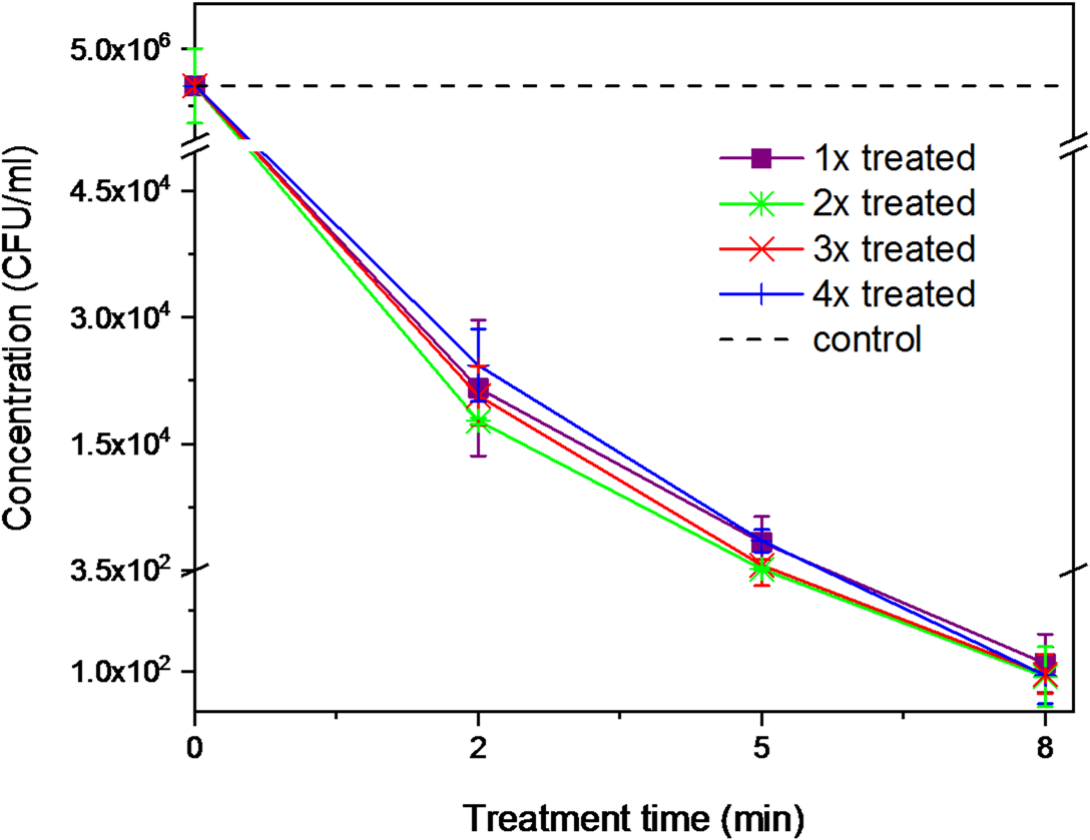
Plasma treatment of S. epidermidis across repeated applications.

**Fig. 14:**
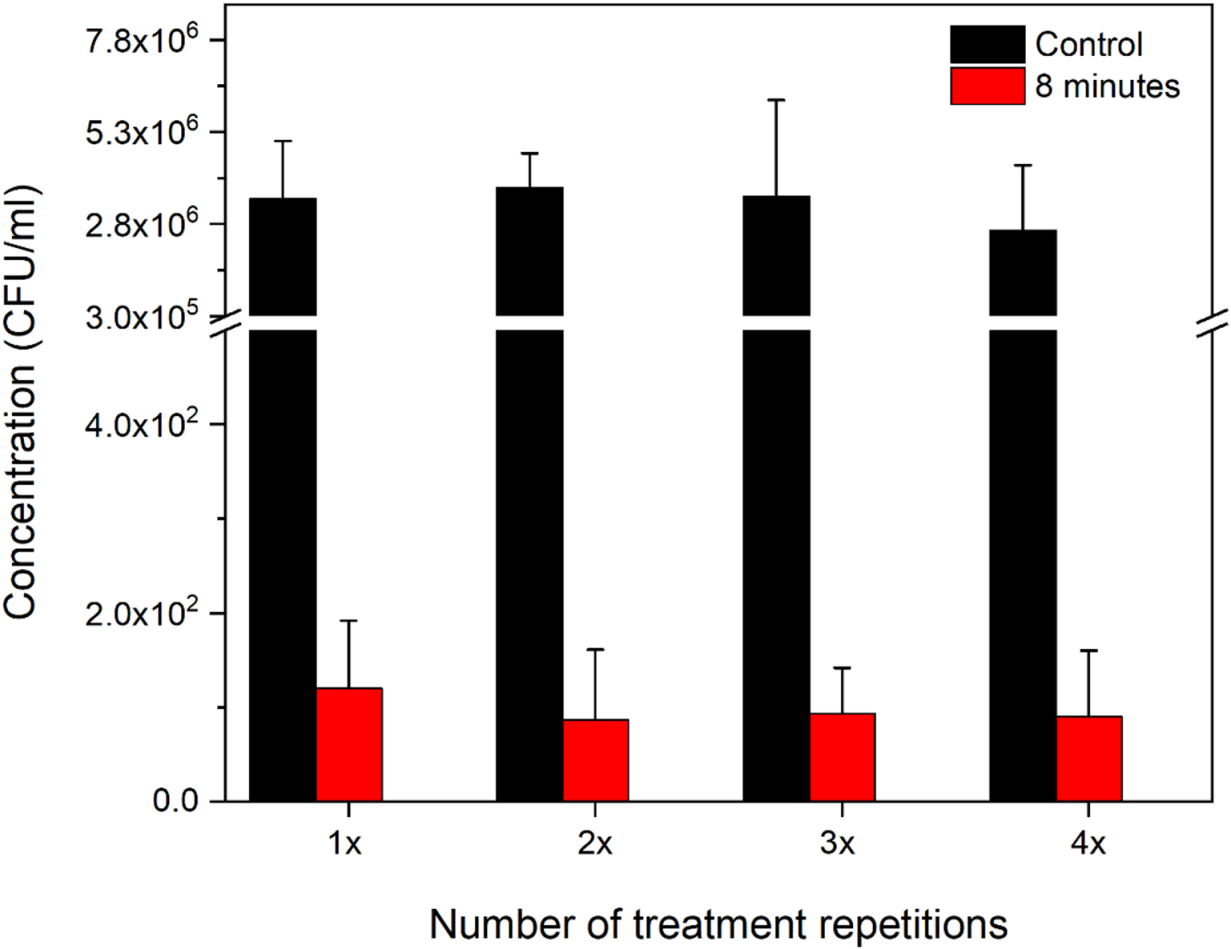
Repeated treatments of S. epidermidis, with a focus on the 8 min treatment time.

A potential development of bacterial resistance to the plasma treatment was also investigated for *S. epidermidis*. In this case, the treatment was repeated four times. After each exposure, a new inoculum was prepared from the previously treated cells and subsequently diluted to the required concentration. A graph illustrating the repeated treatment of *S. epidermidis* is presented in Fig. 13. For clarity, the control is represented by the average initial CFU/mL concentration calculated across all measurements. The individual control values for each repetition are shown in greater detail in Fig. 14, which focuses specifically on the effects of the repeated 8 min treatments.

As shown in Figs. 13 and 14, minor variations were observed between individual repeated applications; however, no significant increase in colony numbers was detected at any of the treatment time (2, 5, 8 min, ANOVA, F (2,6) = 0.03 *p* = 0,988 for all). The detailed logarithmic reduction data supporting this observation are shown in Table 5 in the Appendix. As can be seen, the log reduction increases with treatment duration (from two to eight min) and follows a consistent trend across all four treatment repetitions, with comparable values observed among them. This suggests that, under the tested conditions, even after four consecutive treatments, *S. epidermidis* did not show any indication of developing resistance to plasma exposure.

The antimicrobial efficacy of plasma during the repeated treatments has been demonstrated in previous studies involving both the Gram-positive bacterium *Staphylococcus aureus*^46^ and the Gram-negative *Escherichia coli*^47^. These results further support the potential of plasma technology as a promising alternative for the treatment of infected wounds, with no evidence of the resistance development after multiple exposures.

### 3D movable mechanism potential

The 3D movable plasma torch system enabled precise control over the treatment parameters, such as the movement direction, scanning speed, and repetition. The results showed that more complex movement patterns, such as perpendicular scanning, did not provide any significant advantage over the simpler unidirectional motion. Likewise, an overly rapid scanning reduced efficacy, particularly for the Gram-positive bacteria, due to insufficient exposure time.

In addition, the negligible influence of scanning direction observed in this study is consistent with the geometry of the plasma source used, in which the gas flow is oriented axially towards the treated surface, resulting in an approximately radially symmetric distribution of reactive species. For plasma devices with a similar axial gas-flow configuration, comparable behaviour can be expected. By contrast, systems employing tangential or otherwise asymmetric gas-flow configurations may exhibit directional transport of reactive species along the surface, in which case the scanning direction could play a more prominent role.

Overall, the system allowed for a flexible adjustment of the treatment conditions and demonstrated its potential for use in automated or semi-automated plasma devices, where treatment efficacy, consistency, and adaptability are essential.

#### Consideration of the VBNC state as a limitation

The viable but non-culturable (VBNC) state represents a condition in which bacterial cells remain metabolically active while temporarily losing their ability to grow on standard nutrient media. This state is understood as a stress-induced, reversible survival strategy, where a subpopulation of cells maintains intact membranes, DNA, and basic metabolic functions, yet fails to form colonies under conditions that normally support growth^48^. VBNC induction has been reported under a wide range of environmental stressors, including moderate and long-term physicochemical challenges^48^, and has also been specifically associated with plasma exposure, where reactive species and transient oxidative stress may trigger a shift into this state^49^.

Given that bacteria in the VBNC state do not form colonies but remain viable, their presence may complicate the interpretation of plasma inactivation data. In agreement with previous studies, the return from the VBNC state to a culturable state is possible once the stressor is removed; however, demonstrating resuscitation is experimentally challenging, as renewed growth on agar must be distinguished from pre-existing survivors rather than misinterpreted as recovery of VBNC cells^50^. In the context of our study, the observed post-treatment reductions reflect only the culturable fraction of the population. Therefore, although our results show no evidence of resistance development under the tested conditions, the potential contribution of a VBNC subpopulation cannot be fully excluded and would require targeted assays in future.

## Conclusion

This study examined how scanning direction, scanning speed, and treatment duration influence the antimicrobial performance of a low-temperature microwave plasma torch (Surfayok) against *E. coli* and *S. epidermidis*. The scanning direction had no measurable effect on the decontamination efficiency. This is highly advantageous for practical applications, as it suggests that the plasma source does not require a precisely controlled movement pattern and could be handled manually without compromising efficacy.

Scanning speed showed no statistically significant influence for either microorganism, even though *S. epidermidis* displayed a slight tendency toward reduced inhibition at the fastest movement. Overall, both bacteria responded similarly across all tested speeds. In contrast, treatment duration had a clear and consistent impact. Short exposure times (1–2 min) inactivated *E. coli* more effectively, likely due to its outer cytoplasmatic membrane, whereas longer treatments (5–8 min) resulted in comparable reduction for both species, reflecting the time needed to overcome the more robust Gram-positive cell wall of *S. epidermidis*.

To assess potential medium-related or gas-related effects, additional pH measurements and gas‑only controls were included. The surface pH of both LB and BHI agar showed only minimal changes after plasma exposure, and argon-only controls (8 min) did not differ from untreated plates. These results confirm that the observed antimicrobial effects cannot be attributed to gas flow or acidification and are indeed linked to the plasma itself. Furthermore, colorimetric assays and OES measurements provided qualitative confirmation of reactive species generated during treatment, supporting the mechanistic interpretation of the results.

Repeated-treatment tests further showed no signs of resistance development in *E. coli* or *S. epidermidis* under the tested conditions.

Altogether, these results demonstrate that the 3D-movable plasma system provides a reliable and flexible platform for controlled antibacterial treatment. The independence from scanning direction, the robustness across different scanning speeds, and the clear time-dependent response offer practical advantages for future biomedical applications, where handheld or automated CAP devices could deliver consistent and uniform decontamination.

## Data Availability

The datasets generated and/or analysed during the study are available from the corresponding author on reasonable request.

## Appendix

See Tables 1, 2, 3, 4 and 5.

**Table 1:** Average log reduction (CFU/ml) depending on the treatment direction.

Orientation	Average log reduction [CFU/ml]	Standard deviation
Perpendicular	3,986	0,085
Parallel	4,031	0,054

**Table 2:** Average log reduction (CFU/ml) depending on the treatment time.

	Treatment time [min]	Average log reduction [CFU/ml]	Standard deviation
*E. coli*	1	3,166	0,027
	2	3,874	0,100
	5	4,163	0,130
	8	4,876	0,538
*S. epidermidis*	1	3,017	0,076
	2	3,311	0,075
	5	4,442	0,223
	8	5,729	0,353

**Table 3:** Average log reduction (CFU/ml) depending on the speed of treatment.

	Speed of treatment [mm/s]	Average log reduction [CFU/ml]	Standard deviation
*E. coli*	8,3	3,631	0,448
	16,5	3,769	0,385
	43,0	3,792	0,425
	69,4	3,709	0,496
*S. epidermidis*	8,3	3,229	0,058
	16,5	3,311	0,075
	69,4	3,060	0,069

**Table 4:** Average log reduction (CFU/ml) depending on repeated treatment of E. coli.

	1 × treated	
Treatment time [min]	Average log reduction [CFU/ml]	Standard deviation
2	3,25	0,22
5	3,59	0,38
8	4,00	0,38

	2 × treated	
Treatment time [min]	Average log reduction [CFU/ml]	Standard deviation
2	4,18	0,21
5	4,39	0,36
8	4,54	0,24

**Table 5:** Average log reduction (CFU/ml) depending on repeated treatment of S. epidermidis.

	1 × treated	
Treatment time [min]	Average log reduction [CFU/ml]	Standard deviation
2	2,1967	0,2184
5	3,1755	0,6009
8	4,5003	0,3499

	2 × treated	
Treatment time [min]	Average log reduction [CFU/ml]	Standard deviation
2	2,3099	0,0085
5	3,4564	0,5831
8	4,7619	0,3768

	3 × treated	
Treatment time [min]	Average log reduction [CFU/ml]	Standard deviation
2	2,1219	0,0747
5	3,5228	0,2381
8	4,5218	0,2365

	4 × treated	
Treatment time [min]	Average log reduction [CFU/ml]	Standard deviation
2	1,9617	0,0717
5	2,8256	0,1779
8	4,5352	0,4361
